# ‘A community in crisis’: staff qualitative experiences of NHS and third sector mental healthcare in England

**DOI:** 10.1192/bjo.2025.10826

**Published:** 2025-09-12

**Authors:** Laura Sambrook, Jason C. McIntyre, Rajan Nathan, Jackie Tait, Peter Ashley-Mudie, Matthew Humphreys, Peter Wilson, Pooja Saini

**Affiliations:** Research Associate, School of Psychology, Liverpool John Moores University, UK; Senior Lecturer in Psychology, School of Psychology, Liverpool John Moores University, UK; Forensic Psychiatrist, Cheshire and Wirral Partnership NHS Foundation Trust, Chester, UK; Lived Experience Volunteer, School of Psychology, Liverpool John Moores University, UK; Lived Experience Volunteer, Liverpool John Moores University, UK; Business and Project Manager, Cheshire and Wirral Partnership NHS Foundation Trust, Chester, UK; Consultant Psychiatrist, Cheshire and Wirral Partnership NHS Foundation Trust, Chester, UK; Professor in Suicide and Self-Harm Prevention, School of Psychology, Liverpool John Moores University, UK

**Keywords:** Mental health, third sector, qualitative research, grounded theory, community mental health

## Abstract

**Background:**

More people than ever are receiving support for mental health issues, and instances of suicide continue to grow. Although mental health funding has increased, UK government figures evidence that the National Health Service (NHS) does not have the resources required to respond to such growth in demand. The experiences of staff working in mental health services can offer insight into the efficacy of current provision and assist in service evaluation; however, research examining this issue outside of the COVID-19 pandemic, and in the context of community mental health, is lacking.

**Aims:**

We aimed to explore the perspectives of staff working in a variety of mental health services in North-West England, to elucidate the current standard of care provided and highlight areas for improvement.

**Method:**

One-to-one interviews were conducted with 26 staff members as part of a qualitative grounded theory analysis.

**Results:**

Findings portrayed a community in crisis, consisting of the following themes: stabilisation not recovery, inefficient pathways and barriers to collaboration.

**Conclusions:**

NHS services are struggling to meet the mental health needs of the population, resulting in lengthy waiting times for therapy, a lack of intervention-focused care and an over-reliance on the third sector. While crisis cafés are provided at low cost and result in satisfaction, policy-makers must ensure that these receive adequate funding and do not become overburdened. Staff reported that collaboration between clinical and non-clinical services would improve care pathways and reduce strain on the NHS, but judgemental attitudes and inflexible service development must be challenged to achieve this.

Globally, 970 million people experience mental illness, with one in four affected by a mental disorder at some point in their life.^1^ Considerable disparities in resources exist between countries,^2^ with many allocating little of their overall budget to mental health,^3^ resulting in up to 70% of the population being unable to access treatment.^4^ Although mental health services have undergone a considerable shift from hospital- to community-based care in recent years,^5^ with primary care now serving as the first-level setting for support in high-income countries,^6^ this is not the case for many low- to middle-income countries.^3^ In England, mental health support is provided by a mixture of National Health Service (NHS) organisations, third sector enterprises, local authorities and independent providers. Services can be conceptualised as either primary (mild to moderate), secondary (complex and concurrent) or tertiary (severe and enduring).^7^ Community services play a crucial role in delivering mental healthcare as close to home as possible, while crisis services support individuals requiring urgent support.^8^

Commitments to improve NHS-funded services have been made, and funding for mental healthcare has increased; however, government figures evidence that the number of people unable to access treatment in a timely manner continues to rise, and NHS services do not have the resources they need to respond to such growth in demand.^9^ Although the NHS workforce continues to expand, with around 143 000 people employed to provide mental healthcare,^10,11^ research suggests there are still not enough doctors in mental health services.^12^ Insufficient staffing has been found to directly impact staff morale and service user care,^11^ and those working in community mental health teams (CMHTs) have reported exhaustion and stress due to low staffing levels.^13^ Research suggests that burnout can also manifest in staff working with mentally unwell service users, due to their potential for challenging behaviour and resistance to engaging with support.^14^ In response to these pressures, NHS England^15^ introduced the Community Mental Health Framework and are undertaking subsequent community transformation work, aiming to deliver more person-centred, integrated and accessible care within local communities by coordinating NHS, social care and voluntary sector services through multi-agency teams. While the framework represents a shift towards more holistic and flexible service delivery, the scale of transformation required means that implementation remains ongoing and challenges persist around workforce capacity and timely access.

Crisis cafés and helplines have received considerable investment in recent years, with the aim of reducing pressure on the NHS and providing an alternative to clinical environments such as emergency departments. Crisis cafés are services that provide an informal and accessible setting.^16^ They aim to support anyone in a self-defined crisis and are designed to be accessible at the first signs of crisis, before an individual becomes so unwell that they require in-patient treatment.^17^ They typically operate outside of office hours and allow individuals to enter without an appointment.^16^ They are often led by staff without professional mental health qualifications,^17^ and include volunteers within their workforce.^18^

In comparison, CMHTs continue to face challenges. While the creation of additional specialist mental health services addressed a wide range of needs, it also led to the development of a complicated system in which services are disconnected from one another and psychological interventions are lacking (details available from the author on request). In response to concerns around fragmented community mental healthcare, the care programme approach was introduced, requiring individuals to have a consistent care coordinator and regular treatment reviews in order to promote collaboration and a recovery focus. However, research suggests that, in practice, a lack of coproduction and positive risk taking by care coordinators has been reported.^19^ While the experiences of staff working in CMHTs have been explored,^20^ most recent studies have focused on the impact of the COVID-19 pandemic on well-being^21,22^ rather than capturing staff perceptions of the current standard of mental healthcare in England. There is a paucity of peer-reviewed literature examining the efficacy of crisis cafés and the experiences of their staff, although one recent study suggested that the addition of crisis cafés may be associated with a 7.8% lower hospital admission rate.^23^ Another study examined the attitudes of crisis café managers, finding that factors such as accessibility, relationships with other services and quantity and quality of staff influenced the success of their services.^24^

This study aimed to evaluate the perspectives of staff working in a variety of mental health services in North-West England. We sought to provide insight into their experiences, explore the strengths and limitations of NHS and third sector support and put forward recommendations for improving future practice. It builds upon previous work carried out by the research team^25^ that explored service user experiences of mental health crisis services. This study addresses a gap in the literature, because very few studies have explored staff experiences of working in mental health services without focusing on the COVID-19 pandemic, particularly in relation to community-based mental health services.

## Method

### Design

One-to-one interviews were conducted to explore the experiences of staff working in mental health services, as part of a qualitative grounded theory analysis.^26^ Interviews were semi-structured^27^ to allow for similar questions to be asked of all participants, while allowing for flexibility if pertinent lines of inquiry arose.

### Study setting

The study took place at Cheshire and Wirral Partnership (CWP) NHS Foundation Trust. The Trust provides a range of community and in-patient physical and mental healthcare services, as well as supporting a specific cohort of service users with complex mental health (CMH) needs.

### Ethics statement

The authors assert that all procedures contributing to this work comply with the ethical standards of the relevant national and institutional committees on human experimentation, and with the Helsinki Declaration of 1975 as revised in 2013. All procedures involving human patients were approved by the NHS Health Research Authority and Research Ethics Committee: Integrated Research Application System (IRAS) prior to study commencement (REC ref. 22/EM/0201).

### Participants

The Trust provided the names of 32 members of staff to be interviewed, from 3 CMHTs, 1 crisis line service and 3 crisis cafés. All were deemed suitable by the research team; however, six either declined to take part or did not respond to the invitation. In total, 26 members of staff were interviewed. It was a requirement of the study that participants were employed by a mental health service at the time of interview; however, the nature of participants’ roles varied, ranging from those employed by an NHS Trust to others working as unpaid volunteers in third sector services. Participants were excluded if they were under the age of 18 years or unable to provide written informed consent.

### Materials

Participants were provided with an information sheet and consent form to sign prior to taking part in the study. An interview schedule was developed, with input from relevant stakeholders comprising CWP representatives, public and patient members, commissioners and local authorities (see Table 1). It was designed to facilitate discussions with participants about their experiences of working in mental health teams and the standard of care provided. Prompts were included to guide the discussion, covering areas such as decision-making, therapeutic relationships and current provision.

**Table 1:** Interview schedule questions

Overall topic	Questions
Job role	Can you tell me about your role in terms of main duties and responsibilities?
Job satisfaction	Can you tell me about the areas of your work that you enjoy and areas that you find challenging?
Community mental healthcare	Do you have any thoughts about the care given to patients who are managed under community mental health teams? Are there any issues that make the management of these individuals more challenging?
Crisis mental healthcare	Do you have any thoughts about the care given to patients who attend services in crisis? Are there any issues that make the management of these individuals more challenging?
Decision-making	What influences your decision-making for referring people either to be managed in the community or admitted to hospital?
Short- and long-term goals	How do you balance the long-term goals and immediate needs of service users attending your service?

### Procedures

Staff were identified as eligible to take part in the study, then approached by a member of the research team. Once written consent was provided, interviews were undertaken between February and December 2023. These took place either remotely or in person, either at CWP or third sector premises.

### Data analysis

With the participant’s permission, discussions were audio recorded, transcribed verbatim, checked against the audio files for accuracy by the researcher who conducted the interviews (L.S.) and analysed using grounded theory.^26^ The study adopted an ontologically critical realist approach and was epistemologically objectivist. Participant narratives were accepted as lived realities, even if their accounts simply represented an interpretation of the events they had experienced rather than actualities. The research team adopted a critical reflexive judgement, accepting that behaviours and perceptions are affected by societal norms and expectations. Because some of the authors had personally experienced mental illness (J.T., P.A.-M.) and some had provided mental health support for loved ones or as part of their work (L.S., P.S., M.H.), we embodied a mix of subjective and objective positions. The analysis was both iterative and inductive, with rigour and accurate interpretation of the data maintained through regular meetings between the main analyst and the research team (L.S., P.S., R.N., J.C.M., P.W., J.T., P.A.-M., M.H.). To establish procedural reliability and conceptual credibility,^28^ a member of the research team with experience in qualitative methods examined a sample of transcripts to compare their perceptions of the data with the interpreatation of main analyst (P.S.). Transcripts were hand-coded and subjected to verbatim (line-by-line) coding, followed by descriptive (focused) coding. Next, analytic codes (super-categories) were derived from the merging of descriptive codes and, finally, interpretative codes (themes) were generated from the collapsing, splitting or reorganising of analytic codes. The final theory emerged from the interpretation of the relationship between the themes. Data collection and analysis were carried out concurrently, with the analysis following a participant-by-participant format in which the first transcript was analysed completely before the second, and so on. This ensured that the unique perspectives of each participant were captured, while also examining the commonalities and variations within the sample. The sample size of 26 participants was deemed sufficient for theoretical saturation because our research questions were well defined, our population was relatively homogenous and a rigorous iterative approach to data collection and analysis was taken.

## Results

Just over two-thirds (69%) of the participants identified as female (18/26), and mean age was 40 years (range 24–57 years). At the time of interview, 11 participants worked in CMHTs, four in crisis helplines and 11 worked for, or volunteered at, crisis cafés. The interviews lasted between 20 and 66 min, with an average duration of 39 min (see Table 2). ‘A community in crisis’ was found to be the core category summarising staff experiences, including the following three themes comprising several super-categories: stabilisation not recovery, inefficient pathways for care, and barriers to collaboration (see Table 3). All participant quotes are accompanied by a pseudonym to ensure anonymity.

**Table 2:** Participant demographics

Identifier	Gender	Age (years)	Area of work	Role	Interview duration (min)
Lisa	Female	33	CMHT	Administrative manager	41
James	Male	37	CMHT	Operations manager	55
Peter	Male	56	CMHT	Organisational development	46
Jodie	Female	57	CMHT	Head of clinical services	37
Robert	Male	45	CMHT	Head of clinical services	66
Josie	Female	26	CMHT	Team administrator	26
Donald	Male	45	CMHT	Head of clinical services	34
Daniel	Male	37	CMHT	Project officer	44
Dominic	Male	33	CMHT	Partnerships manager	30
Adrian	Male	38	CMHT	Programme manager	38
Joyce	Female	57	CMHT	Assistant director	30
Marcella	Female	50	Crisis line	Clinical lead	44
Lily	Female	31	Crisis line	First response operative	28
Jasmine	Female	36	Crisis line	Administrator	33
Misha	Female	28	Crisis line	First response operative	35
Lorraine	Female	33	Crisis café	Crisis response worker	20
Janet	Female	30	Crisis café	Crisis response worker	29
Noelle	Female	30	Crisis café	Senior crisis response	43
Jacqueline	Female	24	Crisis café	Service lead	37
Eli	Non-binary	25	Crisis café	Administrator	30
Demi	Female	32	Crisis café	Head of operations	45
Abigail	Female	26	Crisis café	Crisis response worker	47
Gemma	Female	29	Crisis café	Service manager	48
Lydia	Female	57	Crisis café	Crisis response worker	41
Stella	Female	52	Crisis café	Service manager	40
Rachael	Female	26	Crisis café	Senior crisis response	37

CMHT, community mental health team.

**Table 3:** Themes and their corresponding super-categories

Grounded theory analysis: ‘a community in crisis’	
Themes (higher-order themes)	Super-categories (lower-order themes)
Stabilisation not recovery	Lack of provision Crisis management over recovery A need for intervention-focused care Pathologising unhappiness
Inefficient pathways for care	Restrictive criteria for service users to meet before receiving care Unsuitable pathways for individuals with complex needs Siloed (unsupported) working A need for digital interoperability
Barriers to collaboration	Lack of mutual respect between sectors Clinical snobbery Bridging the gap

### Theme 1: stabilisation not recovery

Participants recognised that the mental health services they work within are not meeting the needs of the population, due to staffing issues, extensive waiting lists and a lack of ongoing support (lack of provision). They reported a focus on stabilisation of symptoms rather than rehabilitation (crisis management over recovery), and that collaborative interventions with service users should be implemented (a need for intervention-focused care), but only when referrals are appropriate for mental health teams (pathologising unhappiness).

#### Lack of provision

Twelve participants discussed the current lack of provision for mental health services in North-West England, expressing their disappointment about the standard of care available. One participant described parity of esteem between mental and physical health as ‘nonsense’, while another expressed concern that ‘the people who suffer most are the most vulnerable’. Staff described services as being ’stretched beyond limits’ in a ‘very underfunded system’:

‘We’re not delivering care as we want, in an idealistic model, because we’re so inundated. The structures in place don’t enable culture change.’ – James

Participants highlighted staffing issues as one of their biggest concerns, especially difficulties retaining and recruiting staff. Participants working on crisis helplines described their role as ‘emotionally draining’, resulting in frequent staff sickness. Staff also spoke of extensive waiting lists for therapy and described having been ‘inundated with referrals’ following COVID-19. They discussed the lack of support for individuals awaiting therapy, resulting in increased attendance at crisis cafés and emergency departments in search of help:

‘You’re on this waiting list, but there’s nothing there in the meantime to keep you afloat. It’s either struggle on your own or go to A&E.’ – Josie

Third sector staff acknowledged the strain the NHS is under, highlighting how crisis cafés can provide support in the interim:

‘Because of how thinly resources are stretched, and how much interpersonal care is required, there must be a relationship built up with that person, and many organisations don’t have the time or resources to do that. […] If we didn’t exist, you would see a lot more suicides.’ – Eli

#### Crisis management over recovery

Staff reported a focus on crisis management and stabilisation of symptoms, rather than working towards recovery. They described service users as being ’stuck in a loop’ whereby they access therapy via primary care, receive no ongoing support then require crisis care:

‘We seldom offer any level of hope or recovery because we don’t have any psychological interventions, we don’t have enough staff and all we’re ever doing is crisis management. It’s just medication and nursing. It’s demoralising.’ – Robert

This was concerning for staff, who recognised that defensive practice and risk aversion ‘do not promote what is best for the patient’. Participants described a preoccupation with mitigating risk and admitted that this ‘dominates decision-making’, particularly for staff who have professionally experienced someone completing suicide. They also reported a lack of preventative work in the community:

‘The Trust didn’t previously seem bothered about tackling the problems upstream and trying to do anything preventative. We were waiting for people to come to us at crisis point.’ – Daniel

In contrast, third sector staff predominantly focused on recovery in their work with service users, with nine members of staff working in crisis cafés highlighting future planning, achievable goal setting and signposting as priorities:

‘We want to put the independence back on people and make them in charge of their own recovery; to take that lead of knowing where the support is.’ – Rachael

#### A need for intervention-focused care

Participants felt that CMHTs should be working in a more collaborative manner with service users, focusing on goal setting, psychoeducation and self-management to reduce strain on the system:

‘If you’re coming to a CMHT, you should receive an intervention. There’s no point in you being open to us if you’re just seen once a year. We need to be more proactive in working with a smaller number of people.’ – James

Participants recognised that this was an idealistic view, and that a lack of clinical support and extensive waiting times were barriers to people accessing suitable treatment:

‘People might be out of that critical point by the time they access CBT [cognitive–behavioural therapy]. They might choose not to take part in it any more because they’re like, “I was referred four years ago, what good is that to me now?”’ – Noelle

#### Pathologising unhappiness

Participants reported that situational unhappiness should not require formal intervention. They described an overreliance on the medical model, with some advocating for a more holistic approach. Staff felt that the social determinants of mental health are best addressed early on and that the voluntary sector has a ‘huge role to play’ in proactively addressing issues surrounding debt, work and housing, in the hope that ‘mental health teams can then treat the things that need treating’:

‘We’re getting a lot of referrals where they’re feeling unsatisfied in their job. You don’t need a CMHT or a pill for that. What you need is some time to explore that with a professional who can help you understand your life experiences.’ – James

Third sector staff agreed, acknowledging the importance of signposting to specialist services and listening to service users:

‘We can’t fix 30 years of abuse, low mood and poverty. That sometimes isn’t what they want. They’ll have had counselling; they’ll have had tablets. They just want somebody to say, “Yeah, it is shite”.’ – Marcella

### Theme 2: inefficient pathways for care

Participants felt that a whole-system approach to mental healthcare was required. They discussed inefficient pathways for people with CMH needs, who can be rejected by services for having comorbidities and elevated risk (restrictive criteria), resulting in support being provided by staff who lack appropriate qualifications (unsuitable pathways). Participants felt that the current lack of information sharing (siloed working and a need for digital interoperability) between teams prevents collaboration between services and sectors.

#### Restrictive criteria for service users to meet before receiving care

Seven participants reported concerns about the reluctance of CMHTs to accept referrals for service users with complex needs involving comorbidities or risk:

‘Their focus is around rejecting people from services; they’re looking for reasons not to accept.’ – Donald

Participants discussed a need for ‘culture change’ regarding ‘restrictive’ acceptance criteria for mental health services, to prevent people from ‘falling between gaps’. Staff felt that restrictive criteria were in place to ‘limit risk on organisations’ rather than being in the best interests of service users, with people often rejected from talking therapies services due to reporting suicidal ideation:

‘They go to [service name], have an assessment, say, “Yes, I have thoughts of suicide”. And they go, “Sorry, no”, and then they end up on our [crisis café] front door in suicidal crisis. It’s no longer thoughts of suicide, it’s crisis now because you’ve rejected them.’ – Demi

Third sector staff expressed concerns that, if crisis cafés did not exist, ‘those people would have nowhere to go’ and ‘they would die’. In comparison, crisis cafés were described as ‘very flexible’, with an open-door policy and no time limits on support, although they cannot offer therapy.

#### Unsuitable pathways for individuals with complex needs

Participants discussed the current lack of provision for service users with CMH needs, highlighting extensive waiting lists, a lack of trained staff and stigma as barriers to accessing support. Staff reported that ‘people’s needs have evolved’ and that CMHTs are ill-equipped to support growing numbers of complex individuals:

‘The Complex Needs Team is so saturated. They do such long pieces of work with individuals, so they have a waitlist. While they’re waiting for that service, CMHTs are asked to pick them up, because they are high-risk individuals. But they don’t necessarily have the skills to do that, or to provide meaningful support.’ – Dominic

Third sector staff reported similar concerns in relation to an increase in complex presentations and an inability to offer suitable treatment:

‘We’re stuck – not stuck with them, but we’re playing a waiting game for the actual services they need to pick them up, whilst trying to keep them safe in the meantime.’ – Jacqueline

They expressed frustration that clinical services frequently signpost people with complex needs to crisis cafés, despite their lack of clinical training or access to supervision:

‘We are not trained to be a clinical service. We are not a replacement for a clinical service. But people who are suicidal because of clinical issues are getting left, and that is my worry.’ – Eli

#### Siloed (unsupported) working

Staff discussed the need for more ‘fluid’ referral processes, with one participant highlighting that crisis helpline staff should be able to refer directly into CMHTs. Four participants described their experience of working in mental health teams as ’siloed’ and ineffective:

’Some of the challenges that we’ve had in other services, being commissioned externally, I can’t see anything [organisation name] do. We’re all specialising in our little bits; we’re not globally thinking about the mental health needs of a population.’ – James

Other participants felt that basic in-reach to local primary care networks was lacking, and that a ‘whole-system approach’ was required to identify when service users become unwell in the community to prevent symptoms from escalating:

‘We need to work better as a system. I don’t understand how people end up in crisis without any conversations happening in between, for people who are open to the CMHT. We should have seen that coming 6 months ago, when someone’s starting to deteriorate.’ – Donald

#### A need for digital interoperability

Participants discussed issues around information sharing and data flow, again evidencing siloed working between teams and sectors. NHS staff felt that a shared computer system would be more time effective, whilst third sector staff reflected on their lack of access to the electronic health record, SystmOne, with some believing it would be beneficial for assessment of potential risk:

‘We don’t have any history to gauge with. Someone from crisis line might confidently say, “They’ll be fine”, and they might have insider knowledge of their notes that this is a pattern, whereas we don’t.’ – Janet

Despite this, some felt that not having access to SystmOne reduced their likelihood of unconscious bias resulting from reading about a service user’s history. Several participants felt that the lack of digital interoperability between sectors created barriers to authentic collaboration.

### Theme 3: barriers to collaboration

Most participants wanted more collaborative working between the NHS and the third sector; however, a lack of understanding about what voluntary services can offer (lack of mutual respect between sectors) and stigma towards less traditional practices (clinical snobbery) were identified as potential barriers. Staff recognised that a combination of clinical and non-clinical support could be beneficial in improving care experiences for service users, and in reducing the burden on the NHS (bridging the gap).

#### Lack of mutual respect between sectors

Staff reported that more alliance working and a less ‘transactional relationship’ could be beneficial for providing wrap-around care, with services such as crisis cafés and helplines providing day-to-day, practical support and clinical services providing therapeutic and medical interventions. NHS staff reported ’systemic’ problems in collaborating with the third sector, highlighting ‘a lack of understanding’ and ‘frustration at having to share a budget’ as key issues:

‘I went, “This isn’t right. We’re having to find 20% of the overall spend on the voluntary sector. Why haven’t other areas had to find it?” So, he put that forward, and our contribution to the voluntary sector became smaller, which was brilliant.’ – Robert

Third sector staff described disparities in their relationships with clinical teams, reporting a desire to work closely with them, but expressing that this is difficult because NHS organisations are ’set in their ways’:

‘They will not disclose any information to us, but they’re referring complex clients to us. We’re needing to liaise with them about the complex clients, but we can’t. It makes no sense.’ – Jacqueline

‘We leave voicemails, nothing gets back. It comes across like they don’t care about giving us the information because we’re not a clinical team. But we see that member more than they do. They might see that member once a week, and we see that member every day.’ – Abigail

#### Clinical snobbery

Three participants working in the third sector reported ‘clinical snobbery’ from clinicians with ‘traditional mindsets’. They felt that their opinions were not respected and they found it difficult to ‘have weight’ in discussions about service users. Four participants working for the NHS made similar observations about negative attitudes towards charities within CMHTs:

‘What is the point of the voluntary sector? They can’t give someone an injection. That’s the kinds of perceptions that you’re working with. If you’re not a qualified clinician, you’ve got a much harder time having the credibility than somebody that just, by virtue of being a nurse or doctor, automatically get that respect.’ – Adrian

Despite this, most participants advocated for a combination of clinical and non-clinical support, to achieve ‘variation’ and ‘choice for patients’. They highlighted that non-clinical support can complement clinical work:

‘They do lots of what we do better than we do it. So, why don’t we let them do their bit, and let us do the more complex bit? Instead of trying to pick up everybody in a caseload in a community, why don’t we pick up the ones that actually need it, and work better with our partners?’ – Donald

#### Bridging the gap

The benefits of collaboration were discussed, including cost reductions to the NHS, access to a broader staff skillset, reduced burden on clinical teams and a focus on prevention. Participants wanted to ’streamline partnerships’ to reduce pressure on CMHTs while also recognising the potential benefit for service users:

‘It complements the clinical side of treatment, and it brings people out into the community more.’ – Dominic

Some participants used their lived experience to comment further:

‘As someone who has been through the system, it’s nice to have that fresh set of non-professional eyes on things. It makes all the difference.’ – Jacqueline

Participants felt that it was imperative that everyone understood their role if collaboration was to be successful, highlighting that third sector partners should ‘bridge the gap’ by prioritising preventative work and crisis management over postvention support. It was important to staff that crisis cafés remain non-clinical and continue to promote a flexible alternative to clinical spaces:

‘Clinical teams can’t solve it on their own. Third sector can’t. If we work together, we’ll be doing a bloody good job.’ – Gemma

## Discussion

### Summary of findings

The experiences of staff working in mental health services can offer useful insight into the efficacy of current provision, and assist in service evaluation and improvement. Overall findings portrayed a community in crisis, with participants highlighting inefficient care pathways, a lack of intervention-focused care, risk-averse staff attitudes and a lack of genuine collaboration between the NHS and third sector as factors negatively impacting service user recovery and staff job satisfaction. Participants described mental health services as underfunded and failing to meet population need, with service users becoming increasingly reliant on third sector services such as crisis cafés for support while awaiting therapy. Staff were concerned that some of the most complex individuals are being cared for by those in non-clinical roles, due to extensive waiting lists and a lack of suitably trained NHS staff. Participants felt that genuine collaboration between clinical and non-clinical services would improve care pathways for service users and reduce strain on the NHS; however, barriers in the form of judgemental attitudes and inflexible service development should be challenged to achieve this.

### Comparisons with the wider literature

Staff were disappointed about the current lack of provision for mental health services in the UK. Recent estimates demonstrate that one million people are currently awaiting therapy,^12^ with wait times of more than 6 months for 12% of cases and over 1 year for 6%, resulting in extended periods of time without support.^29^ A survey of 656 people who had attempted to access mental health services within the past 2 years found that 80% experienced a deterioration in their mental health while waiting for support. Of those whose mental health deteriorated, 25% attempted suicide and 42% sought urgent care.^30^ In line with our findings, the evidence base paints a picture of overwhelmed services struggling to provide timely and consistent care, resulting in increased numbers of suicide attempts and a growing reliance on the third sector to provide support in the interim. A lack of parity of esteem was discussed by participants, which is reflected in the literature. Although funding for mental health services has increased, they still receive a disproportionately smaller share of the NHS budget compared with physical health. A survey completed by nursing staff working across a wide range of mental and physical health services in the UK found that 67.9% believed the equality of mental and physical health to be unsuccessful,^31^ in relation to ongoing challenges such as lack of funding, resource allocation and societal attitudes.

Research suggests that even those who receive a mental health intervention are often dissatisfied with the care provided, with service users describing their experiences of therapy as insufficient, brief and lacking follow-up.^30^ This is in line with the views of our participants, who reported a lack of meaningful, collaborative and goal-oriented work carried out in the community. Participants were also concerned about staffing levels, highlighting issues around burnout, retention and training. Although the mental health workforce has seen a steady expansion in numbers since 2017, the rate of growth has been insufficient to meet current demand.^12,13^

Participants voiced concerns about treatment options for those with CMH needs, reporting that current care pathways are not meeting their requirements. They highlighted that clinical services are often reluctant to accept referrals for individuals with comorbidities and elevated risk, with our participants attributing this to self-preservation and lack of resource. Research illustrates that people with CMH needs fall between gaps in services, with one survey finding that 41% of people trying to access treatment felt they were denied support because their condition was not considered sufficiently severe, while 35% said they were denied support because their condition was considered too severe.^30^ This has also been evidenced in the literature on clinician decision-making.^14,32^ Research suggests that, when making decisions, staff hold in mind the possibility of a future imagined investigation into a serious patient safety incident, making a judgement about their practice that could be career damaging,^32^ despite evidence to suggest that engaging with risk-averse practices is countertherapeutic (details available from the author on request). Previous literature has attributed risk-averse decision-making to staff burnout associated with complex diagnoses (details available from the author on request) in relation to understaffing and overstretched services.^14^ Research focusing on service user experiences of care has evidenced that rejection from services can contribute to feelings of resentment and alienation,^33^ resulting in the possibility of losing faith in services and creating patterns of episodic, crisis-driven care.^34^ This was evidenced in our research, where third sector staff reported an increase in complex presentations and feeling ill-equipped to manage the needs of these individuals.

Staff acknowledged the contribution of the third sector towards bridging the gap in service provision and responding to policy initiatives; however, they recognised a knowledge gap concerning its contribution and interface with clinical services. The benefits of third sector support have been recognised within the literature, praising its innovative, accessible and user-defined nature in contrast to the inflexible, risk-averse and biomedical approach of statutory services.^35,36^ In our research, crisis cafés were viewed as a space where new and creative services could be implemented to complement the valuable work undertaken in clinical environments, with an adoption of the recovery model a core aspect of their approach.^37^ This is in line with previous work carried out by the research team, where service users reported that engaging with crisis cafés had contributed to positive treatment outcomes such as reduced psychotropic medication dosage, stronger familial relationships and improved confidence, with several service users stating that crisis cafés had saved their lives.^25^ Third sector spaces have also been praised for being more accessible to those from marginalised groups, such as lesbian, gay, bisexual, transgender, queer or questioning and other sexual orientations and gender identities, and also racialised communities, due to the involvement of ‘lived-experience’ volunteers and the range of support options provided.^38^ Despite this, participants reported ongoing ‘clinical snobbery’ from those working in clinical roles towards third sector staff. Although most participants advocated for a combination of clinical and non-clinical support for service users in the future, it will be important for staff to engage in reflective practice and identify personal biases to avoid further impact on collaboration.

### Strengths and limitations

A strength of the research is the collection of in-depth data from staff working in a variety of mental health services, particularly because research exploring third sector staff experiences is sparse. Access to these individuals provides a close view of reality, with the findings reflecting ‘real-world’ provision. Despite this, the results should be interpreted in the context of some methodological limitations: for example, interviews were conducted at CWP or third sector premises, which may have introduced selection bias or even social desirability bias, because the location of the interviews may have influenced participant responses. Future research may benefit from being conducted in more ‘neutral’ spaces, such as hired university rooms. Another limitation is that our results may not be representative of the rest of the UK (because data were collected only in North-West England), although many issues identified are likely to apply across the country. Additionally, participants’ ethnicity data were not collected which, upon reflection, would have enabled a more nuanced understanding of how experiences and perceptions of care may vary across ethnic groups.

**Fig. 1:**
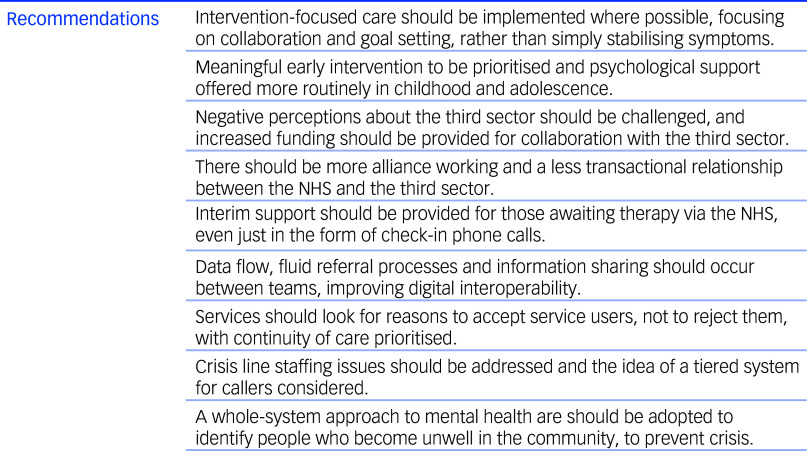
Recommendations for implementation, based on staff interviews. NHS, National Health Service.

This study examined the experiences and perspectives of staff working in mental health services in North-West England, and puts forward their recommendations for future practice (see Fig. 1). Although benefits of both NHS and third sector support were identified, it is evident that NHS services are struggling to meet the needs of the population in a timely manner. There is an expectation that crisis cafés will manage the needs of service users while they await NHS treatment; however, many of these individuals present with CMH needs and require trauma-informed care, which cannot be offered by voluntary staff who are not suitably trained. The NHS Long-Term Plan^7^ concluded that crisis cafés are provided at relatively low cost and result in high satisfaction, which is a sentiment echoed by our participants; however, policy-makers must ensure that the NHS is working together with the third sector on these alternatives, and that they receive adequate funding and do not become overburdened. People accessing mental healthcare should be supported to achieve long-term independence, and our data highlight the fact that third sector organisations are meeting the needs of people who find themselves in crisis; however, these are often small and financially insecure. Ideally, a large organisation would establish a trusted presence in communities and work with lived-experience volunteers to co-produce projects that meet local need, and establish links with other third sector partners as well as clinical services. This would enable mental wellness checks to be offered in community settings, ensure that undeserved communities can access services and cultivate models of care that can help people flourish.

## Data Availability

The data that support the findings of this study are available from the corresponding author, L.S., upon reasonable request.
